# Supplementary material for: Designer spin order in diradical nanographenes
Source: Nat Commun. 2020 Nov 27;11:6076. doi: 10.1038/s41467-020-19834-2 (PMC7695855; doi:10.1038/s41467-020-19834-2)
Supplement: Supplementary file 1 — Supplementary Information [file 41467_2020_19834_MOESM1_ESM.pdf]

Supplementary Information

Designer spin order in diradical nanographenes

Yuqiang Zheng[1†], Can Li[1†], Chengyang Xu[1], Doreen Beyer[2], Xinlei Yue[1], Yan Zhao[1], Guanyong Wang[1], Dandan Guan[1,3], Yaoyi Li[1,3], Hao Zheng[1,3], Canhua Liu[1,3], Junzhi Liu[4], Xiaoqun Wang[1,3], Weidong Luo[1,5], Xinliang Feng[2*], Shiyong Wang[1,3*], Jinfeng Jia[1,3*]

[1]*Key Laboratory of Artificial Structures and Quantum Control (Ministry of Education), Shenyang National Laboratory for Materials Science, School of Physics and Astronomy, Shanghai Jiao Tong University, Shanghai 200240, China.*

[2]*Center for Advancing Electronics Dresden & Department of Chemistry and Food Chemistry, Technische Universität Dresden, 01062 Dresden, Germany.*

[3] *Tsung-Dao Lee Institute, Shanghai Jiao Tong University, Shanghai, 200240, China.*

[4] *Department of Chemistry and State Key Laboratory of Synthetic Chemistry, The University of Hong Kong, Pokfulam Road, Hong Kong, China.*

[5]*Institute of Natural Sciences, Shanghai Jiao Tong University, Shanghai, 200240, China.*

[†]These authors contributed equally to this work.

*Corresponding Authors: shiyong.wang@sjtu.edu.cn, xinliang.feng@tu-dresden.de, jfjia@sjtu.edu.cn

Table of Contents

1. On-surface synthesis of atomically precise nBNGs and BNGs
2. Large-scale STM images
3. Open shell non-Kekulé resonance structures of nBNGs and BNGs
4. DFT calculated spin density distributions
5. Hubbard model calculations of diradical NG dimers
6. Experimental results of other dimer configurations
7. Structural and Magnetic properties of nBNG monomers
8. Hubbard model calculations of an isolated BNG and embedded BNG
9. Perturbation approach simulated line shapes of ferromagnetic and anti-ferromagnetic coupled nanographene dimers.

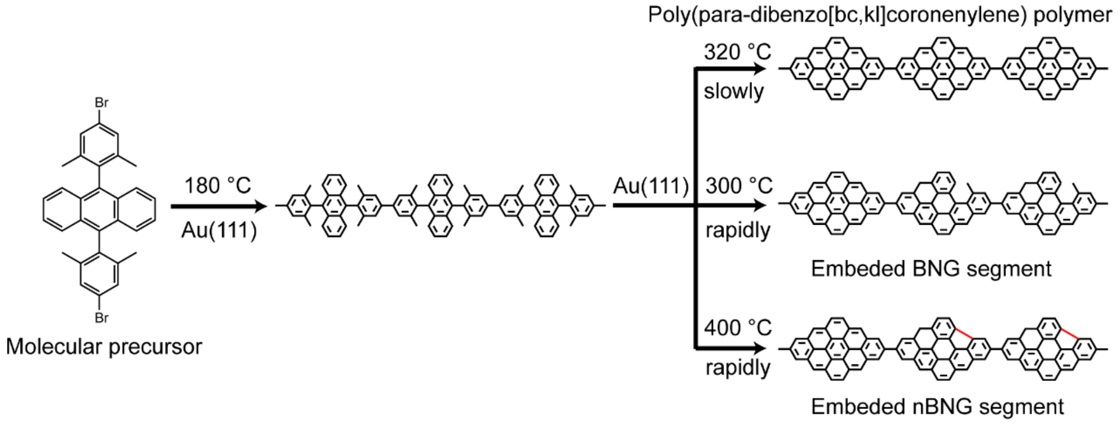

Supplementary Figure 1 | On-surface synthesis of atomically precise nBNGs and BNGs.

The on-surface synthesis method was used to fabricate atomically precise nBNG and BNG by using a molecular precursor of 9,10-bis(4-bromo-2,6-dimethylphenyl)anthracene. A partial zigzag edge terminated poly(*para*-dibenzo[*bc*,*kl*]coronenylene) (PPDBC) polymer can be fabricated by annealing the sample to 180 ^{o}C for 10 minutes to induce polymerizaiton, and then slowly to 320 ^{o}C for the cyclodehydrogenation[1]. Quick annealing the sample to 300 ^{o}C for 5 minutes shall results a partial cyclodehydrogenation, forming partial BNGs. Quick annealing the sample to 400 ^{o}C for 5 minutes shall results a methyl group detachment, forming partial nBNGs.

Using the current molecular precursor, it is a random process for the formation of magnetic NGs, which are embedded in intact PPDBC chains. We didn't observe any isolated magnetic NGs. As revealed by Hubbard model calculations (cf. supplementary Fig. 7), the presence of adjacent PPDBC units has no effect on spin density distribution of magnetic NGs. In addition, the formation of magnetic NG dimer configuration is also random. We didn't observe any uniform magnetic NG oligomers. In order to realize a specific dimer configuration or even uniform magnetic NG oligomer chains, new molecular precursors are required.

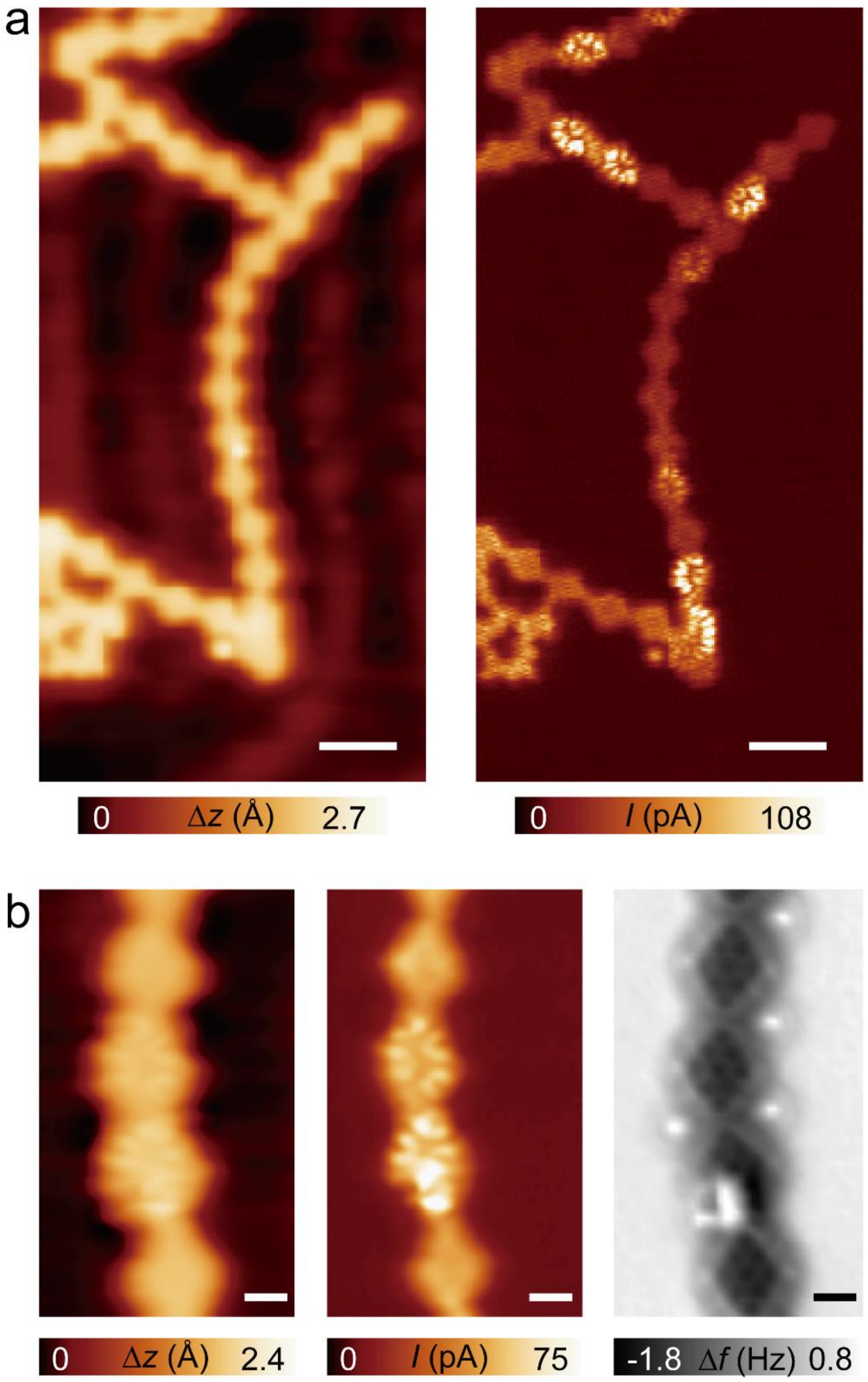

Supplementary Figure 2 | On-surface synthesis of atomically precise nBNGs and BNGs. (a) Large-scale STM image (100mV, 10 PA) and constant-height current image (1mV). (b) Zoomed-in STM image, current image and nc-AFM image showing a nBNG-BNG dimer. Scale bar: (a) 2 nm, (b) 0.5 nm. We found the ratio of BNGs to all units is 17.7%, and the ratio of nBNG to all units is 22.1%. We notice that the population distribution is very sensitive to thermal annealing details. The annealing time and temperature affect the distribution drastically.

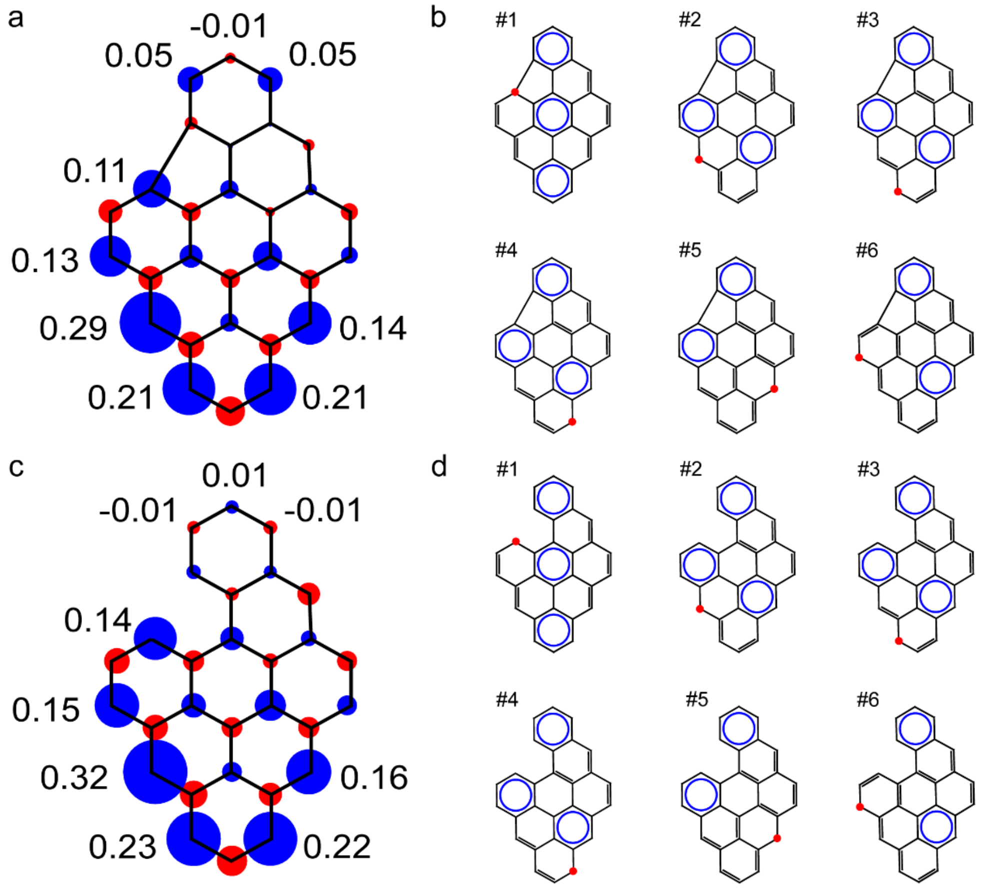

Supplementary Figure 3 | Open-shell non-Kekulé resonance structures of nBNGs and BNGs. a, c, Calcualted spin density distributions of nBNG and BNG respectively. **b, d,** Open-shell non-Kekulé resonances structures of corresponding nBNG and BNG with the blue circles indicating Clar's Sextets. The main features in calculated spin density distribution maps can be qualitatively related to the empirical Clar's Sextet theory[2].

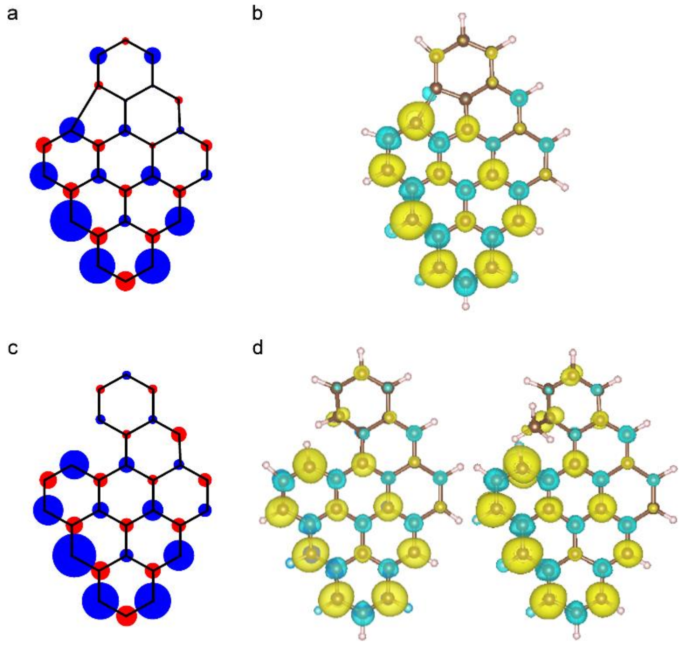

Supplementary Figure 4 | DFT and mean-field Hubbard calculated spin density distribution maps of nBNGs and BNGs.

a, Calculated spin density distributions of a nBNG by mean-field Hubbard model. **b,** Calculated spin density distribution of nBNG by spin-polarized DFT. Blue/red circles denote spin up/spin down density. **c,** Calculated spin density distributions of a BNG by mean-field Hubbard model. **d**, Calculated spin density distributions of BNGs with and without methyl group. The presence of methyl group dos not affect overall spin density distributions. Yellow/aquamarine isosurfaces denote spin up/spin down density.

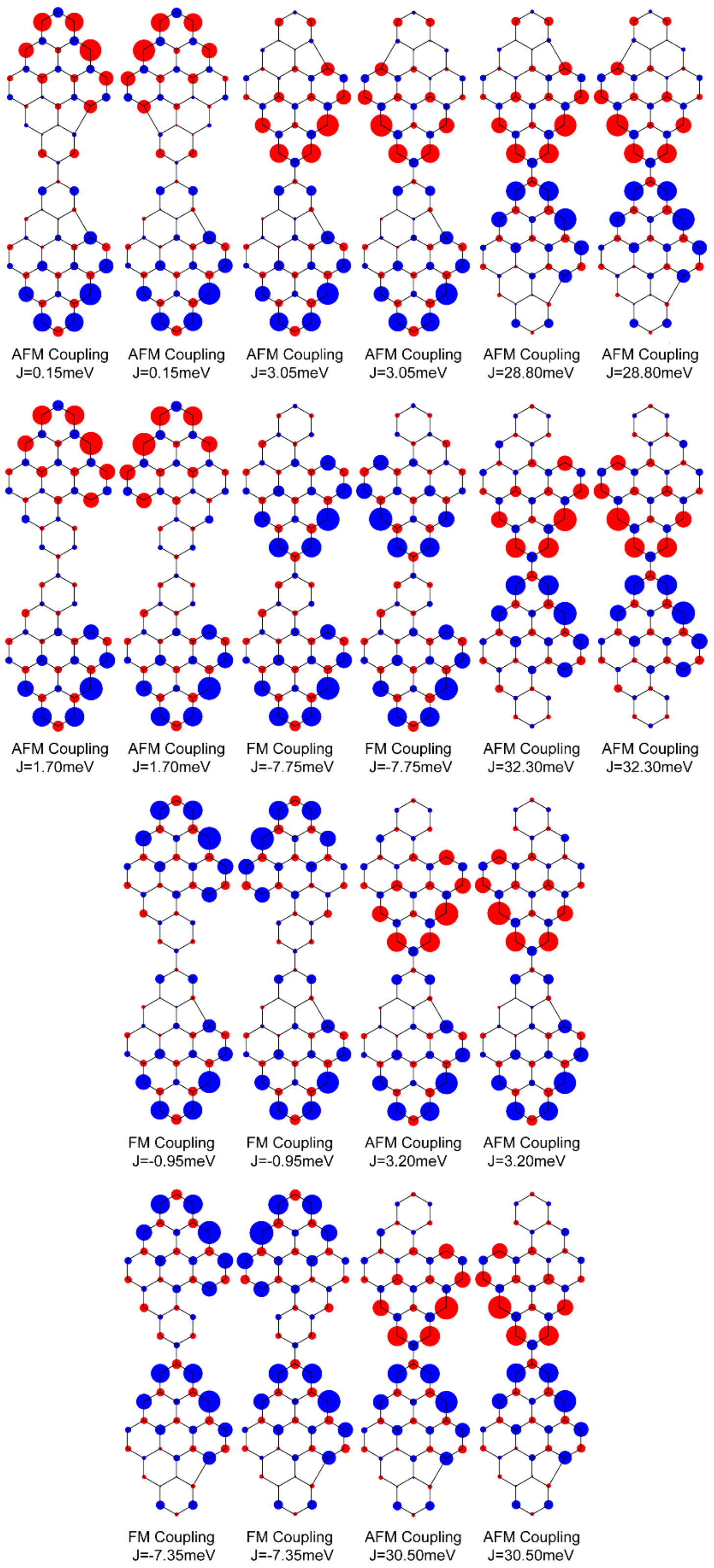

Supplementary Figure 5 | Hubbard model calculations of diradical NGs. Blue/red circles denote spin up/spin down density.

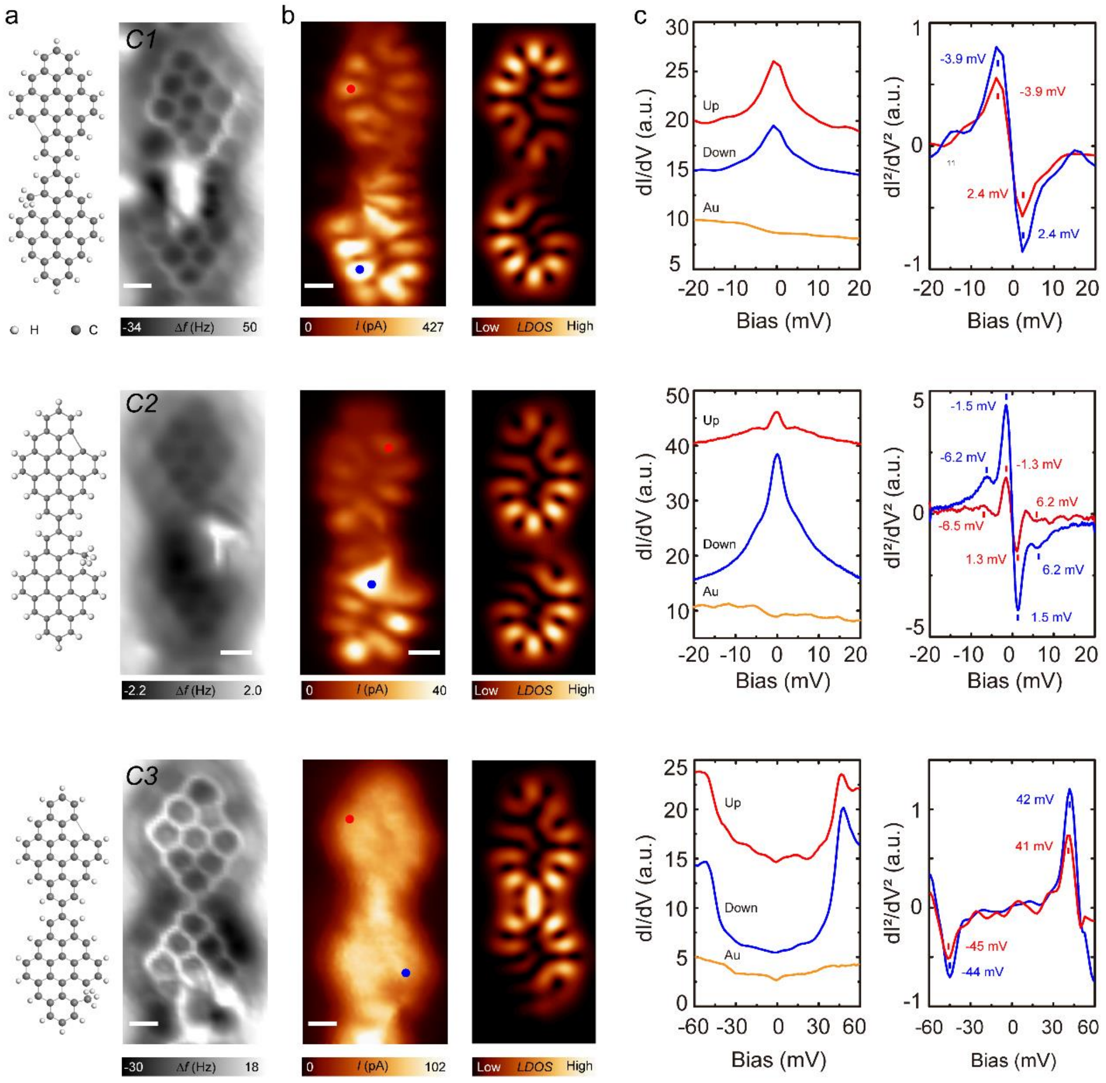

Supplementary Figure 6 | Experimental results of other dimer configurations. a, Chemical structure and nc-AFM image (Resonant frequency: 29 KHz, Oscillation amplitude: 160 pm for C1 and C3, Resonant frequency: 22 KHz, Oscillation amplitude: 140 pm for C2) of BNG-nBNG heterodimers. **b,** Constant-height current image (Bias voltage: 1 mV) and simulated LDOS map of the dimer in **a**. **c,** dI/dV spectra and numerical calculated dI^2/dV^2 spectra taken on locations marked in **b**. A single resonance peak has been detected in C1, indicating two non-interaction spin $S=1/2$, while three resonances have been detected in C2, suggesting a ferromagnetic coupled ground state with $S=1$. The dI/dV spectra in C3 show an anti-ferromagnetic coupling with J=43meV. Scale bars: 0.3 nm.

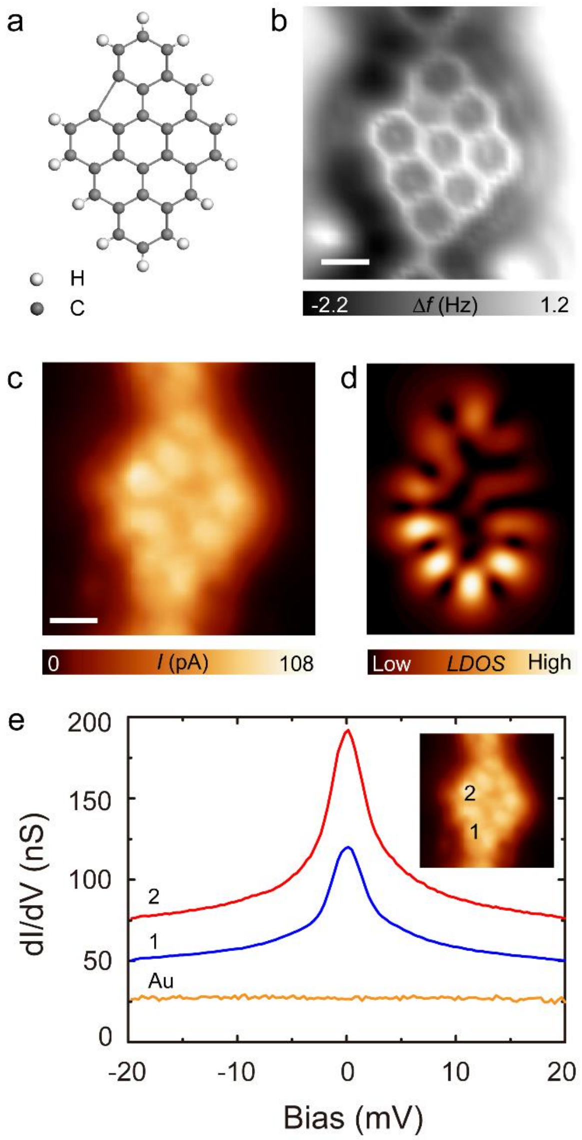

Supplementary Figure 7 | Characterization of a non-bipartite nanographene. **a,** Chemical structure of a non-bipartite nanographene. **b,** Nc-AFM frequency shift image (Resonant frequency: 29 KHz, Oscillation amplitude: 160 pm) of the nanographene in **a**. **c-d,** Constant-height current image (Bias voltage: 1 mV) and simulated LDOS map. **e,** dI/dV spectra taken on the locations marked on the inset current image. A sharp zero-energy peak is resolved due to the screening of the net spin of S=1/2 in nanographene by Au(111) surface electrons. Scale bars: 0.3 nm.

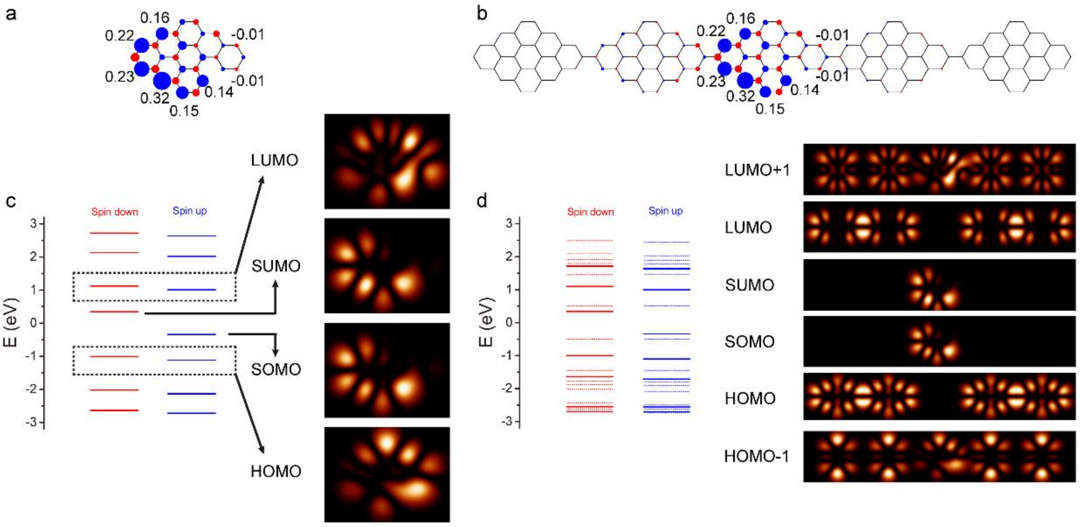

Supplementary Figure 8 | Electronic and magnetic properties of an isolated BNG and an embedded BNG. a-b, Spin density distribution of an isolated and an embedded BNG monomer. **c-d,** Energy spectrum and LDOS maps of an isolated f-NG and an embedded f-NG monomer in a PPDBC chain. The calculations reveal that the presence of adjacent non-magnetic PPDBC segments does not affect the magnetic properties of the embedded magnetic NGs.

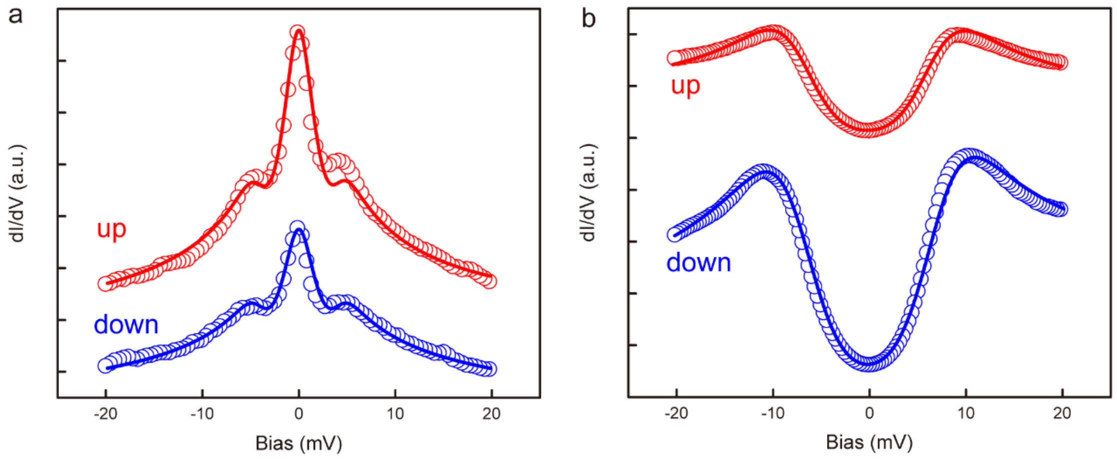

Supplementary Figure 9 | Perturbation approach simulated line shapes of ferromagnetic and anti-ferromagnetic coupled nanographene dimers. a, d*I*/d*V* spectra (circles) and perturbation method fitted line shapes of a diradical NGs with *S=1*. Fitting parameters:$J\rho_0 = -1.37$; ω_0=7; T_0^2=3.5; *b*=0.001; V_{off}=0; σ_0=1.25; V_R=19.8485. **b,** d*I*/d*V* spectra (circles) and perturbation method fitted line shapes of a diradical NGs with *S=0*. Fitting parameters: $J\rho_0 = -1.56$; ω_0=81.2; T_0^2=4.3; *b*=0.03; V_{off}=-0.034; σ_0=1.2; V_R=20.157. The $J\rho_0$ means the magnetic exchange strength between net magnetic moment in diradical NGs with the itinerant electrons of substrate; the σ_0 indicates the sample electron bandwidth, which affects the full width half maximum of Kondo peak in the sample. The T_0^2 is the tip-sample interaction strength, *b* is the background of dI/dV slope coming from the tip or the substrate. The σ_0 represents the additional background conductance value. The V_{off} and V_R are bias offset values and ranges of simulated dI/dV line shapes, respectively.

To understand the scattering process in STM junction, we fitted our magnetic field dependence spectra using the perturbation approach up to third order developed by Ternes[3,4]. Although this perturbation approach only works in weak coupling regime (weak coupling between magnetic impurity and electron reservoir) and will give a line shape with FWHM independent of temperature, it can effectively capture the STM tunneling process, giving a straight forward ling shape of tunneling spectra. We fitted our spin-flip spectra of ferromagnetic and anti-ferromagnetic coupled dimers. The spectra can be reproduced with a scattering strength $J\rho_0$= -1.37 and -1.25 for upper and lower halves of ferromagnetic C2 BNG dimer. Besides, the perturbation theory suggests its ferromagnetic exchange interaction strength is -4meV by the fitting lines (the effective temperature is 5K in this method). On contrary, the fitting parameters

of anti-ferromagnetic coupled hetero-dimer are $J\rho_0$=-1.55 and -1.07 for upper and lower halves of the anti-ferromagnetic dimer and anti-ferromagnetic coupling is 6.6meV(the effective temperature is 5K in this method). The good agreement between the fitted line shapes and experimental spectra indicate the observed zero peaks at fermi level could originate from Kondo resonance for S=1 due to the scattering of magnetic impurity by surface electrons, and U gap with 7meV between fermi level stems from spin flip for the anti-ferromagnetic spin exchange S=0 in two spin systems.

Supplementary References

1. Beyer, D. *et al.* Graphene Nanoribbons Derived from Zigzag Edge-Encased Poly(para-2,9-dibenzo[bc,kl]coronenylene) Polymer Chains. *J. Am. Chem. Soc.* **141**, 2843–2846 (2019).
2. Clar, E. *Polycyclic Hydrocarbons*. (Springer-Verlag Berlin Heidelberg, 1964).
3. Ternes, M. Spin excitations and correlations in scanning tunneling spectroscopy. *New J. Phys.* **17**, 063016 (2015).
4. Ternes, M., Heinrich, A. J. & Schneider, W.-D. Spectroscopic manifestations of the Kondo effect on single adatoms. *J. Phys. Condens. Matter* **21**, 053001 (2009).
